# Excellent Intra- and Interobserver Reliability of the D.H. Dejour Version 2 Classification for Trochlear Dysplasia Using Radiographs Combined With MRI With Evaluation of Shortcomings

**DOI:** 10.1177/23259671251395739

**Published:** 2026-01-26

**Authors:** Michael J. Dan, Nicolas Cance, Edoardo Giovannetti de Sanctis, Tomas Pineda, David Henri Dejour

**Affiliations:** † Lyon Ortho Clinic, Orthopedic Surgery Department, Clinique de la Sauvegarde, Lyon, France; ‡ Macquarie University, Sydney, Australia; § Lingard Hospital, Merewether, Australia; ‖ IULS- Institut Universitaire Locomoteur et Sports, Pasteur 2 Hospital, CHU, Nice, France; Institution performed at Lyon Ortho Clinic, Orthopedic Surgery Department, Clinique de la Sauvegarde, Lyon, France

**Keywords:** patellar instability, Dejour classification, reliability, knee surgery, sport medicine

## Abstract

**Background:**

The D.H. Dejour classification (Version 2 [V2]) expanded upon the H. Dejour radiographic classification of trochlear dysplasia by adding computed tomography (CT) scans to the evaluation. Magnetic resonance imaging (MRI) then became the main investigation of choice.

**Purpose:**

To report the reliability of the Dejour V2 using a combination of radiographs and MRI instead of CT scan as per the original classification and to explore differences in the assessment of trochlear dysplasia between assessors to better understand limitations to the classification.

**Study Design:**

Cohort study; Level of evidence, 3.

**Methods:**

This is a retrospective comparative study, conducted by reviewing a prospectively maintained institutional database, between 2 groups of patients: those with recurrent patellar dislocation, termed objective patellar instability (OPI), and control patients with no patellofemoral symptoms. Inclusion criteria were available preoperative imaging including both knee MRI and a true lateral view radiograph of the knee at 20° of flexion and no history of previous knee surgery. Imaging evaluation was performed independently by 2 orthopaedic surgeons, and each trochlea was classified according to the Dejour V2 classification. To classify, all reviewers initially used the lateral radiograph, then confirmed with MRI slice imaging.

**Results:**

A total of 200 patients were included in the statistical analysis (OPI, n = 123; control, n = 77). In the control group, 13% of patients presented with trochlear dysplasia type A, whereas 87% of patients had a normal trochlea. The kappa coefficient was 0.77 for intrarater reliability and 0.75 for interrater reliability, representing a substantial level of agreement. In the OPI group, 97% of patients presented a trochlear dysplasia. The kappa coefficient was 0.92 for intrarater reliability and 0.86 for interrater reliability, representing an excellent correlation between reviewers. When simplified from 4 types to 2 types of trochlear dysplasia, high-grade (supratrochlear spur present) versus low-grade (no supratrochlear spur present), the intrarater reliability and interrater reliability improved to 0.95 and 0.93, respectively, and there was a 97.8% sensitivity and 96.4% specificity for diagnosing high-grade trochlear dysplasia.

**Conclusion:**

Utilizing radiographs and MRI for the Dejour V2 classification of trochlear dysplasia, we demonstrated only moderate sensitivity in diagnosing low-grade trochlear dysplasia utilizing the 4 types of trochlear dysplasia. The sensitivity for diagnosing low-grade trochlear dysplasia, along with the overall intra- and interrater reliability was improved by simplifying the classification from 4 types of dysplasia to 2 grades, low versus high grade, based on the presence of a supratrochlear spur.

Trochlear dysplasia is present in 96% of patients with recurrent patellar dislocations, termed objective patellar instability (OPI).^12^ It was originally classified into 3 types by Henri Dejour, in 1990, based on the level of the intersection (crossing sign) of the trochlear floor with the lateral and medial condyles on true lateral knee radiographs.^11^ In 1998, David H. Dejour then expanded the original classification into 4 types by cross-referencing true lateral radiographs with 2-dimensional computed tomography (CT) scan slice imaging adding 2 signs: the supratrochlear spur and the double contour.^6^

The main criticism made to the D.H. Dejour classification (Version 2 [V2]) is a low inter- and intraobserver reproducibility on magnetic resonance imaging (MRI) assessment,^20,25^ which can be improved when simplified to high- versus low-grade dysplasia.^16,26^ Consequently, simplified classifications have been proposed, such as the Oswestry-Bristol Classification (OBC).^22^ However, such a classification is based on only an axial evaluation of the trochlear shape and therefore fails to evaluate the sagittal trochlear offset, known as the supratrochlear spur, which requires the sagittal images to be reviewed. When radiographs are not well performed, or not incorporated in the evaluation of trochlear dysplasia with multislice CT or MRI imaging, this has been shown to be associated with worse reliability when assessing the classification of trochlear dysplasia.^19^

The importance of classifying trochlear dysplasia is to identify OPI patients who might benefit from a deepening trochleoplasty. It has been shown that patients with a supratrochlear spur, whether classified as Dejour type B or D, might benefit from a deepening trochleoplasty with improved patient-reported outcomes and low rates of long-term arthritis.^7,10,13^ This surgical treatment is not indicated in patients without an increased prominence/supratrochlear spur (type A or C).

While the need to perform a trochleoplasty is debated, trochleoplasty in combination with a medial patellofemoral ligament reconstruction (MPFL-R) results in a lower redislocation rate when compared with an isolated MPFL-R alone, respectively having a redislocation rate of 2% and 7%.^1^ Furthermore, an isolated MPFL-R without a trochleoplasty has been shown to be associated with worse clinical outcomes in patients with a supratrochlear spur. This supports the clinical importance of trochleoplasty^15^ and highlights the need for analysis of the sagittal plane to allow measurement of the supratrochlear spur in the assessment of patellofemoral patients.

We hypothesize that the principles of the D.H. Dejour classification V2 that utilized a combination of radiographs and CT scans can be transferred to MRI with good reliability in trained assessors. The aim of this research was therefore to report the reliability of the D.H. Dejour classification V2 using a combination of radiographs and MRI, instead of CT scan as per the original classification. The secondary aim was to explore differences in the assessment of trochlear dysplasia, providing insights for a more comprehensive future MRI classification of trochlear dysplasia with improved observer reliability.^8^

## Methods

### Ethics

All patients provided informed consent for the use of their data for research, and the study was approved by the ethical board in advance.

### Study Design

This is a retrospective comparative study conducted at the Lyon Ortho Clinic (Lyon, France) referral center for patellar instability, reviewing a prospectively maintained institutional database. Two groups of patients have been compared: group 1 was patients with documented patellar dislocation and group 2 was a control group of patients with no patellofemoral symptoms.

Patients within the institutional database with a documented diagnosis of OPI between 2020 and 2022 were identified. Patellar dislocation was diagnosed and noted by the senior author (D.H.D.) at the time of the consultation. Group 2, the control group, consisted of meniscal injury patients without patellar instability or pain and no ligamentous injury or past surgical history.

Inclusion criteria were available preoperative imaging, including a knee MRI and a true lateral view radiograph of the knee at 20° of flexion and no previous knee surgery. Standard MRI on a 1.5-T magnet. T2-weighted MRI slices with multiplanar reconstruction (MPR) were employed. All measurements were conducted using the MPR mode. MPR^14^ allows for simultaneous displays in the axial, sagittal, and coronal planes of the acquired knee images (3-dimensional sequence acquisition of an anatomic volume). The MPR mode enables standardization of knee rotation in the axial plane to ensure true lateral views (independent of patient position during the scan) by aligning the sagittal plane perpendicular to the posterior bicondylar line. This axial plane could then be used to reconstruct the 2 orthogonal planes—that is, sagittal and coronal—with the knee in its manipulated orientation. Correct orientation of the MRI planes is akin to ensuring a true lateral radiograph.

Patients with knee osteoarthritis, visible growth plates, or inappropriate preoperative lateral knee radiographs or MRI were not included in the study. Radiographs were considered inappropriate if there was not perfect (>1 mm) overlap/superimposition of the femoral condyles. The MRI was considered adequate if fat-saturated T2-weighted axial slices were visible from the upper to the lower part of the trochlea, to classify the trochlear dysplasia.

### Classification

**Figure 1.**
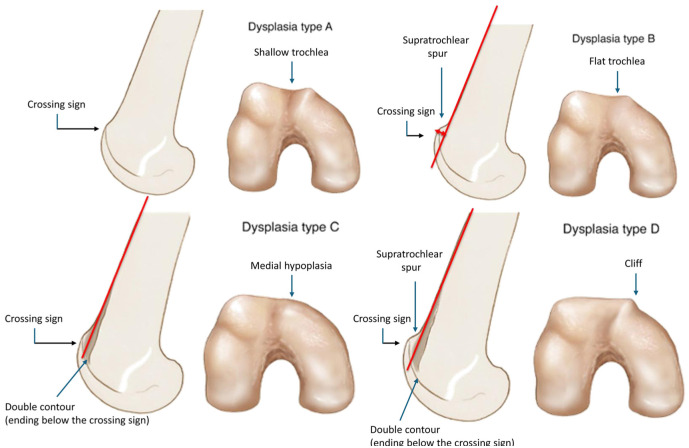
Dejour classification for trochlear dysplasia.

The assessment of trochlear morphology was performed retrospectively and independently by 3 orthopaedic surgeons (2 trained assessors M.J.D. and N.C., and 1 senior surgeon D.H.D.), using an open source image viewer Horos (Horos Project). Each trochlea was classified according to the classification described by Dejour et al V2 (Figure 1).^6^ All reviewers evaluated initially the true lateral radiograph and then the MRI slice imaging. To reduce bias, OPI and control groups were randomly analyzed, using deidentified Digital Imaging and Communications in Medicine, and the evaluation was performed twice (4 weeks apart) to measure intraobserver reliability.

### Statistical Analysis

The intraobserver/interobserver method of agreement was determined using the Cohen kappa statistic. The kappa statistic expresses the chance-corrected agreement. It is the (normalized) observed agreement minus the agreement expected on the basis of chance alone. The expected agreement is based on the prevalence of each type, which was calculated from the combined ratings of all raters. Six levels of agreement were retained depending on the values of kappa (k): excellent (0.81 < k < 1), substantial (0.61 < k < 0.8), moderate (0.41 < k < 0.6), fair (0.21 < k < 0.4), slight (0.0 < k < 0.2), and poor (k < 0).^24^ The other 2 assessors were individually compared with the senior author for interrater reliability, and the mean Cohen kappa was presented.

## Results

A total of 200 patients were included in the study based on the criteria outlined above: 123 patients in the OPI group (no patient was excluded from the analysis) and 77 patients in the control group.

Pooled results demonstrated there was excellent intra- and interrater reliability of 0.95 and 0.90, respectively, with excellent sensitivity and specificity (Table 1).

**Table 1:** Reliability for Dejour Version 2 Normal and 4 Grades of Dysplasia

	Intrarater Reliability	Interrater Reliability
Overall	0.95	0.90
Control group	0.77	0.75
Objective patellar instability patients	0.92	0.86

In the control group, 13% of patients presented with type A trochlear dysplasia, whereas 87% of patients had a normal trochlea. The kappa coefficient was 0.77 for intrarater reliability and 0.75 for interrater reliability, representing a substantial level of agreement (Table 1).

In the OPI group, 97% of patients presented with some form of trochlear dysplasia. The kappa coefficient was 0.92 for intrarater reliability and 0.86 for interrater reliability, representing an excellent correlation between reviewers (Table 1). A breakdown of the types of trochlear dysplasia is presented in Table 2.

**Table 2:** Incidence of Trochlear Dysplasia in Objective Patellar Instability*^*a*^*

Dysplasia Type	Incidence (n = 123)
Normal	4 (3)
A	19 (15)
B	33 (27)
C	8 (7)
D	59 (48)

a Data are presented as percentages.

When simplified to high- (B or D) versus low-grade (A or C) trochlear dysplasia, intra- and interrater reliability were improved for both pooled results and individually in the control and OPI cohorts (Table 3).

**Table 3:** Reliability for Dejour Version 2 Normal Versus High (B+D) Versus Low (A+C) Grade of Dysplasia

	Intrarater Reliability	Interrater Reliability
Overall	0.95	0.93
Control group	0.84	0.82
Objective patellar instability patients	0.92	0.88

The sensitivity of the Dejour 4 grade classification was less than optimal for Dejour type A and B, only 65.5% for type A and only 90.9% for type B (Table 4). This was improved by moving to the 2 classifications of trochlear dysplasia, with low grade having a sensitivity of 83.8% and high grade 97.8% (Table 5).

**Table 4:** Sensitivity and Specificity for Dejour Version 2 Normal and 4 Grades of Dysplasia*^*a*^*

	Normal	A	B	C	D
Sensitivity	98.6	65.5	90.9	100	98.3
Specificity	96.2	98.2	97.0	99.5	99.3

a Data are presented as percentages.

**Table 5:** Sensitivity and Specificity for Dejour Version 2 Normal Versus High (B+D) Versus Low (A+C) Grade of Dysplasia^*a*^

	Normal	Low Grade	High Grade
Sensitivity	98.6	83.8	97.8
Specificity	98.5	98.2	96.4

a Data are presented as percentages.

When examining just the OPI patients, there was a 97% sensitivity and 96% specificity in diagnosing trochlear dysplasia as high grade. In OPI patients, disagreement between observers in classifying patients as Dejour V2 type A or B occurred. Given type B differs from type A by the presence of a supratrochlear spur, we measured the spur of the patients where this disagreement occurred. The spur measured on the radiograph averaged 1.75 mm in these patients where there was disagreement as Dejour V2 type A or type B.

## Discussion

The most important finding of this study was an excellent interobserver reliability between orthopaedic surgeons, with a kappa coefficient of 0.95 and 0.90, respectively, for intra- and interrater reliability in the pooled control cohort and OPI population. Furthermore, simplifying the classification from 4 to 2 types of trochlear dysplasia, the reliability was improved to 0.93, with excellent sensitivity (97.8%) and specificity (96.4%) demonstrated for diagnosing high-grade trochlear dysplasia (type B or D). This demonstrates that the use of MRI, instead of CT, in combination with radiographs is a validated form of assessment of trochlear dysplasia.

Our results demonstrated excellent specificity but only moderate sensitivity of 70%, for diagnosing trochlear dysplasia type in the control cohort compared with normal. This is reflected again in our pooled results of both the control cohort and the OPI, where the sensitivity for diagnosing type A dysplasia was only 65.5%. This means we were very accurate at ruling out trochlear dysplasia, but our ability to detect trochlear dysplasia could be improved in low-grade trochlear dysplasia. Utilizing a quantitative measurement, such as the sulcus angle^4^ or lateral inclination^3^ to quantify the trochlear shape, might improve the sensitivity of diagnosing low-grade trochlear dysplasia.

One of the main issues with the Dejour classification is its nonlinear severity progression: types B and D are considered high-grade trochlear dysplasia; type C, despite its alphabetical location, is considered a lower grade of trochlear dysplasia than type B. Erroneously, several papers evaluating the Dejour V2 classification have tried to simplify the grading system into high- versus low-grade dysplasia, but often include type C with types B and D, with resultant poor reliability.^17,18^ Type C was not meant to be included in high-grade dysplasia as part of the original classification and may reflect too much complexity in the classification due to the nonlinear progression of severity. High-grade trochlear dysplasia refers to the presence of a supratrochlear spur (types B and D), whereas type C does not have a supratrochlear spur, and as a result, our results suggest any new classification should focus on the presence of the supratrochlear spur. Low-grade trochlear dysplasia has not been shown to benefit from a deepening trochleoplasty.^5,7,13^ The clinical need to identify the supratrochlear spur has been shown through worse patient-reported outcome scores in those patients treated with an isolated MPFL-R who had a supratrochlear spur compared with those who did not have a spur.^15^ This demonstrates the importance of identifying the spur with respect to clinical outcomes and suggests a trochleoplasty could potentially improve their outcome.^6,8,13,15^ and highlights that the focus of classifying trochlear dysplasia should be on identifying whether a supratrochlear spur is present.

We had excellent sensitivity and specificity in detecting high-grade trochlear dysplasia when using the simplified 2 types of classification (type B or D) of 97.8% and 96.4%, respectively, whereas the sensitivity for type B dysplasia was 90.9%, suggesting we would miss 9.1% of patients who may benefit from a trochleoplasty. This again highlights how the classification can be improved by focusing on identifying and quantifying the supratrochlear spur in the classification of trochlear dysplasia. A quantitative measurement of the spur, with a quantitative cutoff in future classifications, would be of benefit to improve agreement about the presence of a supratrochlear spur, which is of clinical relevance.

The use of MRI now exceeds that of CT scans, on which the Dejour V2 classification was based. We have demonstrated good-to-excellent reliability with the use of multislice MRI imaging for the Dejour V2 classification. However, novel MRI classifications attempt to simplify the classification of trochlear dysplasia. The OBC attempts to classify trochlear dysplasia based on MRI,^22^ grouping patients in a 4-part classification system comprising normal, mild, moderate, and severe to represent a normal, shallow, flat, and convex trochlea, respectively. The OBC is based on axial slice imaging alone,^22^ like Brattstom^2^ did on axial view in 1964. While it shows improved inter- and intraobserver reliability compared with the Dejour V2 classification, it only utilizes the axial slices and therefore fails to identify the supratrochlear spur, which we believe is the most important determinant of high-grade trochlear dysplasia and needed to propose a treatment algorithm.^9^ Clinical implementation of the OBC classification, however, reported no recurrent instability, but its use has not been examined outside the founding institution.^23^ The 2-part classification by Yang et al^27^ will differentiate a Dejour C from a D, but fails to identify a Dejour type B, which is considered high-grade dysplasia and would benefit from a trochleoplasty.^1^ Other studies have demonstrated poor reliability when only axial images are used for classification.^21^ Therefore, it is our opinion that classifications of trochlear dysplasia should include multiplane slice imaging to accurately diagnose and treat trochlear dysplasia.

The present study highlighted a good-to-excellent inter- and intraobserver reliability using both lateral radiographs and MRI axial slice imaging in trained assessors. To improve reliability in untrained surgeons and facilitate the classification of trochlear dysplasia using MRI only, both axial and sagittal slices would be needed.

### Limitations

Limitations of this study include the fact that patients in our control population were not completely asymptomatic, as they had a meniscal injury; however, no link has been demonstrated between OPI and meniscal injury, and the control population had no patellofemoral symptoms, thus making the present control population as close to a normal population as possible. Also, we only report on radiographic outcomes, focusing exclusively on the reliability of the Dejour V2 classification for trochlear dysplasia using MRI.

A novel adaptation of the Dejour V2 classification to MRI aims to address some of the limitations of V2 identified in our paper.^8^ In the current paper, we demonstrated poor sensitivity in diagnosing low-grade trochlear dysplasia (type A). The novel MRI adaptation of the classification, Dejour Version 3 (V3), utilizes either the sulcus angle or lateral trochlear inclination to improve the sensitivity for diagnosing low-grade trochlear dysplasia, known as type 1 in V3. In our study, the reliability was improved when moving from 4 to 2 grades of trochlear dysplasia; this is reflected in V3 of the classification, where type 3 is considered high-grade trochlear dysplasia, determined by the presence of a supratrochlear spur >5 mm.^8^

## Conclusion

Utilizing radiographs and MRI for the Dejour V2 classification of trochlear dysplasia, we demonstrated only moderate sensitivity in diagnosing low-grade trochlear dysplasia utilizing the 4 types of trochlear dysplasia. The sensitivity for diagnosing low-grade trochlear dysplasia, along with the overall intra- and interrater reliability, was improved by simplifying the classification from 4 types of dysplasia to 2 grades, low versus high grade, based on the presence of a supratrochlear spur.
